# Metabolic adaptability and nutrient scavenging in *Toxoplasma gondii*: insights from ingestion pathway-deficient mutants

**DOI:** 10.1128/msphere.01011-24

**Published:** 2025-04-02

**Authors:** Patrick A. Rimple, Einar B. Olafsson, Benedikt M. Markus, Fengrong Wang, Leonardo Augusto, Sebastian Lourido, Vern B. Carruthers

**Affiliations:** 1 Department of Microbiology and Immunology, University of Michigan Medical School 12266 , Ann Arbor, Michigan, USA; 2 Whitehead Institute for Biomedical Research, Cambridge, Massachusetts, USA; 3 Department of Pathology and Microbiology, and Immunology, University of Nebraska Medical Center, Omaha, Nebraska, USA; University at Buffalo - Downtown Campus, Buffalo, New York, USA

**Keywords:** *Toxoplasma gondii*, CRISPR, metabolism, amino acids

## Abstract

The obligate intracellular parasite *Toxoplasma gondii* replicates within a specialized compartment called the parasitophorous vacuole (PV). Recent work showed that despite living within a PV, *Toxoplasma* endocytoses proteins from the cytosol of infected host cells via a so-called ingestion pathway. The ingestion pathway is initiated by dense granule protein GRA14, which binds host endosomal sorting complex required for transport (ESCRT) machinery to bud vesicles into the lumen of the PV. The protein-containing vesicles are internalized by the parasite and trafficked to the plant vacuole-like compartment (PLVAC), where cathepsin protease L (CPL) degrades the cargo, and the chloroquine resistance transporter (CRT) exports the resulting peptides and amino acids to the parasite cytosol. However, although the ingestion pathway was proposed to be a conduit for nutrients, there is limited evidence for this hypothesis. We reasoned that if *Toxoplasma* uses the ingestion pathway to acquire nutrients, then parasites lacking GRA14, CPL, or CRT should rely more on biosynthetic pathways or alternative scavenging pathways. To explore this, we conducted a genome-wide clustered regularly interspaced short palindromic repeats (CRISPR) screen in wild-type (WT) parasites and Δ*gra14*, Δ*cpl*, and Δ*crt* mutants to identify genes that become more fitness conferring in ingestion-deficient parasites. Our screen revealed a significant overlap of genes that potentially become more fitness conferring in the ingestion mutants compared to WT. Pathway analysis indicated that Δ*cpl* and Δ*crt* mutants relied more on pyrimidine biosynthesis, fatty acid biosynthesis, tricarboxylic acid (TCA) cycle, and lysine degradation. Bulk metabolomic analysis showed reduced levels of glycolytic intermediates and amino acids in the ingestion mutants compared to WT, highlighting the pathway’s potential role in host resource scavenging. Interestingly, Δ*cpl* and Δ*crt* showed an exacerbated growth defect when cultured in amino acid-depleted media, suggesting that disrupting proteolysis or the export of proteolytic products from the PLVAC affects parasite survival during nutrient scarcity.

**IMPORTANCE:**

*Toxoplasma gondii* is an obligate intracellular pathogen that infects virtually any nucleated cell in most warm-blooded animals. Infections are asymptomatic in most cases, but people with weakened immunity can experience severe disease. For the parasite to replicate within the host, it must efficiently acquire essential nutrients, especially as it is unable to make several key metabolites. Understanding the mechanisms by which *Toxoplasma* scavenges nutrients from the host is crucial for identifying potential therapeutic targets. Our study suggests that the ingestion pathway contributes to sustaining parasite metabolites and parasite replication under amino acid-limiting conditions. This work advances our understanding of the metabolic adaptability of *Toxoplasma*.

## INTRODUCTION

The obligate intracellular parasite *Toxoplasma gondii* infects up to 30% of the world’s human population (1). This success expands beyond humans as *Toxoplasma* can infect any nucleated cells in most warm-blooded animals. The extremely large host range suggests that *Toxoplasma* has evolved versatile mechanisms to support its growth.

**Fig 1:**
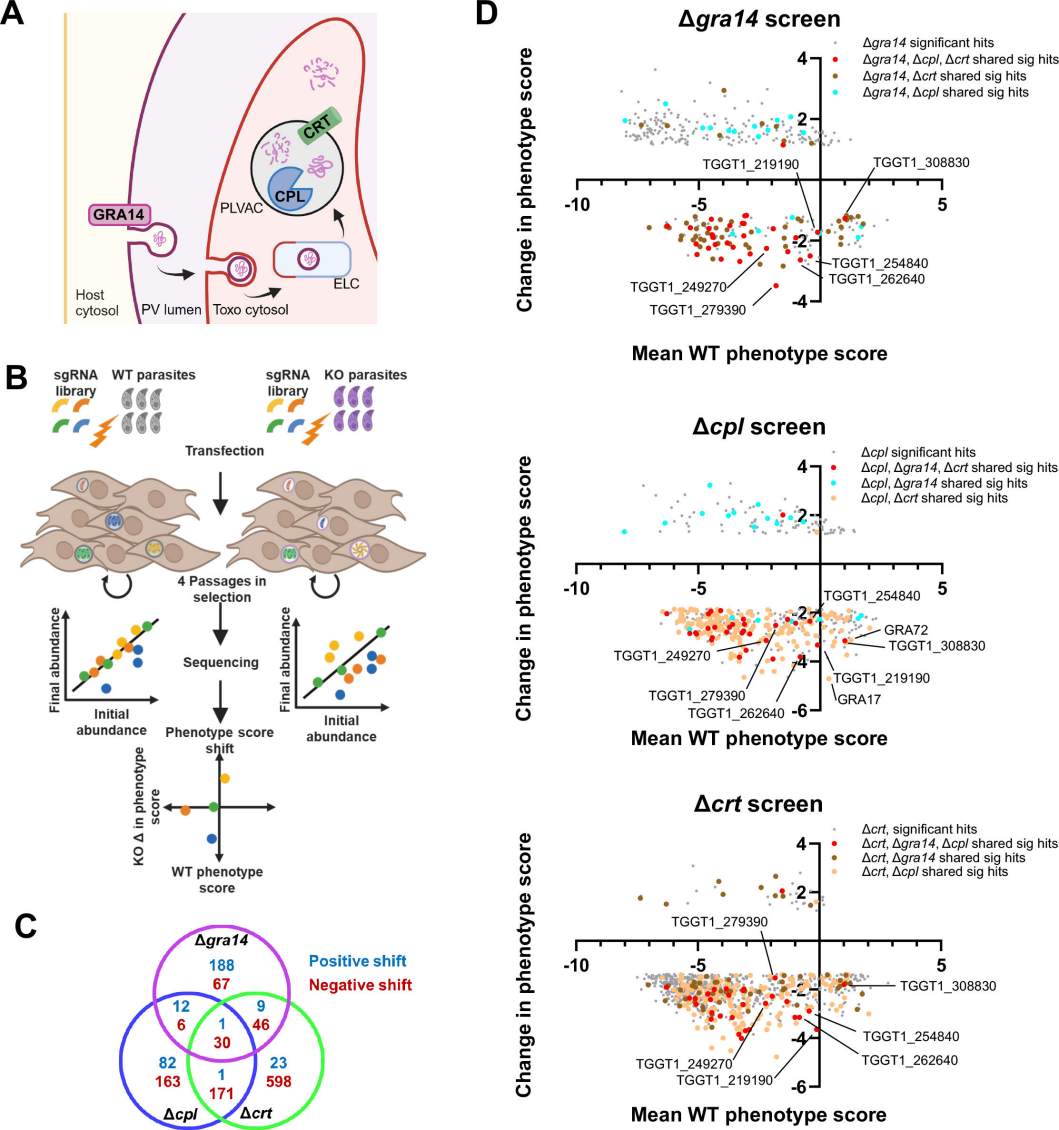
Genome-wide CRISPR screen identifies fitnessconferring genes related to the ingestion pathway. (A) Schematic of the ingestion pathway wherein GRA14 is at the PVM to facilitate uptake of the host cytosolic protein. The ingested material is trafficked through the parasite’s endosome-like compartment (ELC), delivered to the PLVAC for degradation by CPL and liberated by CRT. (B) Schematic of the CRISPR screens. Parasites (6 × 10^8^) were transfected with a linearized plasmid library containing 10 sgRNAs per gene of the *Toxoplasma* genome. Parasites were passed four times in pyrimethamine to select for integration of the sgRNA-expression cassettes and subsequent disruption of the gene. sgRNA abundance was determined from the initial library and final population through Illumina sequencing. The phenotype score was calculated based on the top five sgRNAs for each gene. (C) Venn diagram indicating significant hits shared between samples. Cyan indicates genes shifted positively; red indicates genes shifted negatively. (D) Graphs depicting the significantly shifted hits for each screen, with the change in phenotype score on the *y* axis and the phenotype score for the wild-type (WT) screen on the *x* axis. Gray indicates hits pertaining to just the mutant on the graph; red indicates sharing between all mutants; cyan indicates sharing between Δ*gra14* and Δ*cpl*; tan indicates sharing between Δ*cpl* and Δ*crt*; and gold indicates sharing between Δ*gra14* and Δ*crt. n* = 3 biological replicate screens for each strain.

Like other intracellular pathogens, *Toxoplasma* derives its nutrients from the host cell it infects (2). This is achieved through a combination of scavenging (3, 4) and host cell manipulation (5) to complement *de novo* biosynthesis (6). To scavenge material from the host cell, *Toxoplasma* must bypass the parasitophorous vacuole membrane (PVM), which protects the parasite from intrinsic host defenses (7). Scavenging of small soluble metabolites is facilitated by diffusion across the PVM through the nutrient pore proteins GRA17, GRA23, GRA47, and GRA72 (8–11). Once in the parasitophorous vacuole (PV), transporters on the parasite plasma membrane mediate uptake into the parasite cytosol for utilization (12–14). Lipids are scavenged by sequestering host-derived vesicles within the PV and transfer of lipid material to the parasite by unknown mechanisms (15–17). Another potential nutrient acquisition route for *Toxoplasma* is the ingestion pathway, which entails parasite uptake of host cytosolic material. Using the secretory protein GRA14, *Toxoplasma* co-opts the host’s endosomal sorting complex required for transport (ESCRT) machinery at the PVM to generate vesicles that the parasite internalizes, likely at a specialized site of endocytosis, the micropore (18–20). The ingested material traffics through the endosome-like compartment toward the plant vacuole-like compartment (PLVAC) (21). Once inside the PLVAC, the ingested proteins are degraded by TgCPL (22), the major protease of the PLVAC (23, 24), and the digested products are likely liberated by a small peptide and amino acid transporter, the chloroquine resistance transporter (TgCRT) (25, 26) (Fig. 1A). Despite previous observations demonstrating that the ingestion pathway facilitates the acquisition of host cytosolic proteins, the exact reason for its employment by *Toxoplasma* remains unknown, particularly because disrupting this pathway has limited impact on parasite fitness *in vitro* (25).

We hypothesize that in the absence of the ingestion pathway, *Toxoplasma* compensates by tapping into alternative nutrient and metabolite sources such as through upregulation of biosynthesis pathways or increased utilization of other nutrient uptake systems. To test this, we employed a genome-wide clustered regularly interspaced short palindromic repeats (CRISPR)-mediated gene-disruption screen to identify genes that become more fitness conferring in ingestion-deficient mutants compared to wild-type (WT) parasites. By analyzing mutants that are deficient in three different steps of the pathway (Δ*gra14*, Δ*cpl*, and Δ*crt*), we aimed to uncover genes and pathways that compensate for the loss of ingestion, thereby shedding light on the broader metabolic adaptability and nutrient acquisition strategies of *Toxoplasma*.

## RESULTS

Before performing a genome-wide CRISPR screen to identify genes that are differentially fitness conferring in ingestion mutants, we first generated ingestion mutant strains in an RHCas9 strain background. We transfected RHCas9 parasites (27) with sgRNAs targeting the first exon of *GRA14*, *CPL*, or *CRT* to generate loss-of-function insertion or deletion (INDEL) mutants. We verified the disruption of *GRA14*, *CPL*, and *CRT* by Sanger sequencing across the sgRNA-targeted sites (data not shown) and western blotting (Fig. S1A through C). We also confirmed with an ingestion assay that RHCas9Δ*gra14* parasites have reduced uptake of host cytosolic protein (Fig. S1D).

A schematic of the screen is presented in Fig. 1B. Briefly, we screened the WT, Δ*gra14*, Δ*cpl*, and Δ*crt* strains using an established protocol (28) with minor modifications, including passaging the library of mutant parasites four times instead of three times to give more time for fitness mutants to drop out of the population. After the final passage, genomic DNA was extracted and used to PCR amplify sgRNAs for sequencing and comparison to the sgRNAs in the initial library. We calculated the phenotype score for each gene in WT or mutant parasites by determining the log2 fold change in relative abundance from the initial sgRNA pool to the final population. To minimize the effects of a bottleneck, we performed this analysis only for the top five most abundant sgRNAs per gene.

A modest bottleneck occurred in our screens, with our final libraries (P4) missing about 30%–60% of the sgRNAs (Fig. S2A). Part of this bottleneck was probably due to random chance since the dispensable genes, those with neutral or positive selection, on average retained 8 of 10 sgRNAs per gene, which was significantly smaller than the input of 10 sgRNAs at P0 (*P* < 0.05 for WT and *P* < 0.01 for mutants). The essential genes, those that undergo negative selection, on average retained only 3 of 10 sgRNAs, which was significantly different from the input and the dispensable genes (*P* < 0.0001 for all samples), suggesting that the loss of sgRNAs for the essential genes was due to both random loss and phenotypic selection (Fig. S2B through D). Despite the modest bottleneck, phenotype scores for the WT screen had an *R*² value of 0.85 compared to the original CRISPR screen in this strain (29), which is comparable or higher than other published screens (30, 31) (Fig. S2E). We also observed a high correlation among WT replicates, suggesting reproducibility (Fig. S2F). Additionally, previously known essential and dispensable genes were stratified along a phenotype score of −2.5, with essential genes having lower scores and dispensable genes having higher scores, consistent with previous screens (29) (Fig. S2G).

After calculating the phenotype scores, we assessed for each gene whether the shift in the phenotype score between WT and mutants was statistically significant (Table S1). We used a set of 497 genes expressed only in the sexual stages of the parasite as a reference cohort of dispensable genes to account for random variation in phenotype scores in WT and mutant parasites (32). We applied two criteria for significance: first, identifying genes with a shift greater than 2 SDs from the mean shift among the sexual stage genes, and second, performing a Wilcoxon signed-rank test using the shift in sexual stage genes as the control group to identify significantly shifted genes (false discovery rate [FDR] < 0.01). Genes meeting both criteria were labeled as significantly shifted.

This analysis identified 149, 307, and 845 genes with significantly lower phenotype scores in Δ*gra14*, Δ*cpl*, and Δ*crt* mutants, respectively, and 210, 96, and 34 genes with significantly higher phenotype scores, respectively (Fig. 1C and D). We looked for hits that are shared among all three mutants and found 30 genes that become significantly more fitness conferring in all three mutants compared to WT (Table 1).

**TABLE 1:** Shared genes from Δ*gra14*, Δ*cpl*, and Δ*crt* fitness-conferring hits^*a*^

Gene ID	Product description	LoPIT	WT mean phen score	Δ*gra14* mean phen score	Δ*cpl* mean phen score	Δ*crt* mean phen score	Sidik et al. (29) phen score
TGGT1_308830	Dual specificity protein phosphatase	N/A	1.01	−0.26	−2.14	−0.78	−0.17
TGGT1_219190	Nuclear movement protein domain-containing protein	Cytosol	−0.11	−1.83	−3.43	−3.75	−1.68
TGGT1_254840	Tetratricopeptide repeat-containing protein	N/A	−0.42	−2.92	−2.77	−3.31	−1.4
TGGT1_262640	ApiCox23	Mitochondrion (membranes)	−0.82	−3.46	−4.62	−3.97	−3.49
TGGT1_221320	Acetyl-CoA carboxylase ACC1	Apicoplast	−1.01	−2.91	−3.44	−4.15	−3.6
TGGT1_286530	ApiCox24	Mitochondrion (membranes)	−1.34	−3.7	−3.62	−3.84	−2.82
TGGT1_279390	Proliferation-associated protein 2G4, putative	Nucleus (non-chromatin)	−1.82	−5.31	−4.33	−3.35	−0.88
TGGT1_288880	Hypothetical protein	N/A	−1.94	−3.55	−5.84	−4.22	−4.6
TGGT1_249270	Protein disulfide isomerase-related protein (provisional), putative	Apicoplast	−2.21	−4.47	−5.35	−4.78	−4.56
TGGT1_235398	Hypothetical protein	Mitochondrion (soluble)	−2.89	−5.32	−5.76	−6.54	−4.79
TGGT1_267560	Folate-binding protein YgfZ	Mitochondrion (soluble)	−3.03	−4.21	−6.57	−6.73	−3.34
TGGT1_254135	Folate-binding protein YgfZ	N/A	−3.11	−4.29	−5.58	−5.09	−2.94
TGGT1_220940	Ribosomal RNA methyltransferase (FtsJ) family protein	N/A	−3.13	−4.57	−5.58	−5.76	−2.38
TGGT1_313140	Isocitrate dehydrogenase	Mitochondrion (soluble)	−3.15	−4.99	−5.41	−5.18	−3.04
TGGT1_254530	Hypothetical protein	N/A	−3.2	−4.63	−6.04	−7.21	−4.69
TGGT1_273840	Brix domain-containing protein	Nucleus (non-chromatin)	−3.31	-6	−7.13	−7.16	−3.18
TGGT1_319930	Hypothetical protein	Nucleus (chromatin)	−3.58	−5.8	−6.07	−6.74	−3.76
TGGT1_227970	Histone family DNA-binding protein	Apicoplast	−3.72	−5.22	−6.3	−5.95	−2.15
TGGT1_254800	Hypothetical protein	Mitochondrion (soluble)	−3.83	−5.91	−6.63	−5.8	−3.51
TGGT1_218770	Carrier superfamily protein	Mitochondrion (membranes)	−4.08	−6.68	−5.99	−7.17	−4.06
TGGT1_271400	Hypothetical protein	N/A	−4.32	−6.45	−6.92	−6.32	−4.91
TGGT1_233930	CAF1 family ribonuclease	Mitochondrion (soluble)	−4.42	−6.31	−7.44	−7.38	−4.53
TGGT1_267570	Hypothetical protein	N/A	−4.45	−6.25	−7.56	−7.67	−4.14
TGGT1_243780	Hypothetical protein	Nucleus (chromatin)	−4.48	−5.77	−6.41	−6.89	−4.68
TGGT1_229620	Hypothetical protein	Mitochondrion (soluble)	−4.58	−6.18	−6.62	−6.76	−4.47
TGGT1_243440	Histone lysine acetyltransferase GCN5-B	Nucleus (chromatin)	−4.6	−6.85	−7.23	−6.78	−2.96
TGGT1_261070	Apicoplast triosephosphate translocator APT1	Apicoplast	−5.07	−7.53	−7.94	−7.68	−4.44
TGGT1_236210	Peptidase M16 family protein, putative	Mitochondrion (membranes)	−5.1	−7.21	−7.92	−7.47	−4.74
TGGT1_256970	Vacuolar ATP synthase subunit A, putative	PM (peripheral 2)	−5.34	−7.21	−8.19	−7.7	−3.61
TGGT1_205470	Translation elongation factor 2 family protein, putative	Cytosol	−6.29	−7.81	−8.33	−8.2	−4.31

^*a*^ N/A, not available; phen, phenotype.

To determine whether the genes identified as more fitness conferring in the mutants were true hits, we tested six genes (TGGT1_308830, TGGT1_219190, TGGT1_254840, TGGT1_262640, TGGT1_279390, and TGGT1_249270) from among those identified in all three mutants, particularly those with phenotype scores of >−2.0 in WT parasites indicating knock-out feasibility. WT, *Δgra14*, or *Δcpl* parasites were transfected with sgRNAs targeting the six genes. sgRNAs targeting *sag1* and *cdpk1* were included as negative and positive controls, respectively (33). Nascent transfected populations were directly plaqued in the presence of vehicle (ethanol, no drug selection) or 3 µM pyrimethamine (drug selection) for 7 or 10 days, respectively. The wells were fixed, stained with crystal violet, and imaged. Using a modified Cellpose pipeline, we automated measuring the number and size of plaques (Fig. S3A). The no-drug selection wells served as a correction factor to determine the number of viable parasites within drug selected wells.

As expected, the *Δcdpk1* wells produced few to no plaques since *CDPK1* is an essential gene (33). This result confirms that disrupting fitness-conferring genes reduced the WT and mutants’ fitness. There were trends toward Δ*gra14*Δ*254840* and Δ*cpl*Δ*254840* having lower plaque efficiencies than WTΔ*254840* (Fig. S3B), but the differences were not statistically significant. Compared to Δ*sag1*, WTΔ*262640* plaques were statistically smaller, while there was a similar trend in the Δ*gra14*Δ*262640* plaque size. Additionally, Δ*247290* parasites trended toward smaller plaque sizes in both WT and Δ*gra14* parasites. The smaller plaque size in the WT strain for these mutants suggests these genes are false positives (Fig. S3C). Notably, in previous screens, the phenotype scores of Δ*262640* and Δ*247290* were lower (−4.0 and −4.8, respectively) than what we observed (−0.8 and −2.2, respectively). None of the other genes had significantly smaller plaque sizes, indicating an inability to conclusively identify synthetic lethal genes. However, we verified that disrupting fitness-conferring genes reduced the WT and mutant fitness.

We re-examined our data to assess patterns in the ingestion mutant pairs. The largest overlap in significant genes was between Δ*cpl* and Δ*crt* with a total of 201 genes, likely due to both proteins being in the PLVAC. Thus, some hits could be involved in compensating for phenotypes associated only with the PLVAC. The Δ*gra14* and Δ*cpl* mutants and the Δ*gra14* and Δ*crt* mutants shared 76 and 36 genes, respectively. This suggests these genes probably do not function together in other pathways outside the ingestion pathway (Fig. 1C).

To determine if this overlap was significant or due to random chance, we tested the hypergeometric overlap and found a significant overlap in all three gene pairs. This led us to conclude that our CRISPR screens successfully identified genes that were more fitness conferring due to the disruption of the ingestion pathway or the PLVAC.

To identify potential compensatory mechanisms among the more fitness-conferring genes, we conducted a metabolic pathway analysis using tools available on ToxoDB. No clear compensatory pathway emerged from the 30 genes that conferred higher fitness and were shared by all three mutants (Table S2). Thus, we broadened our search to include pathway pairs to determine if a higher number of genes could reveal more fitness-conferring metabolic pathways.

**Fig 2:**
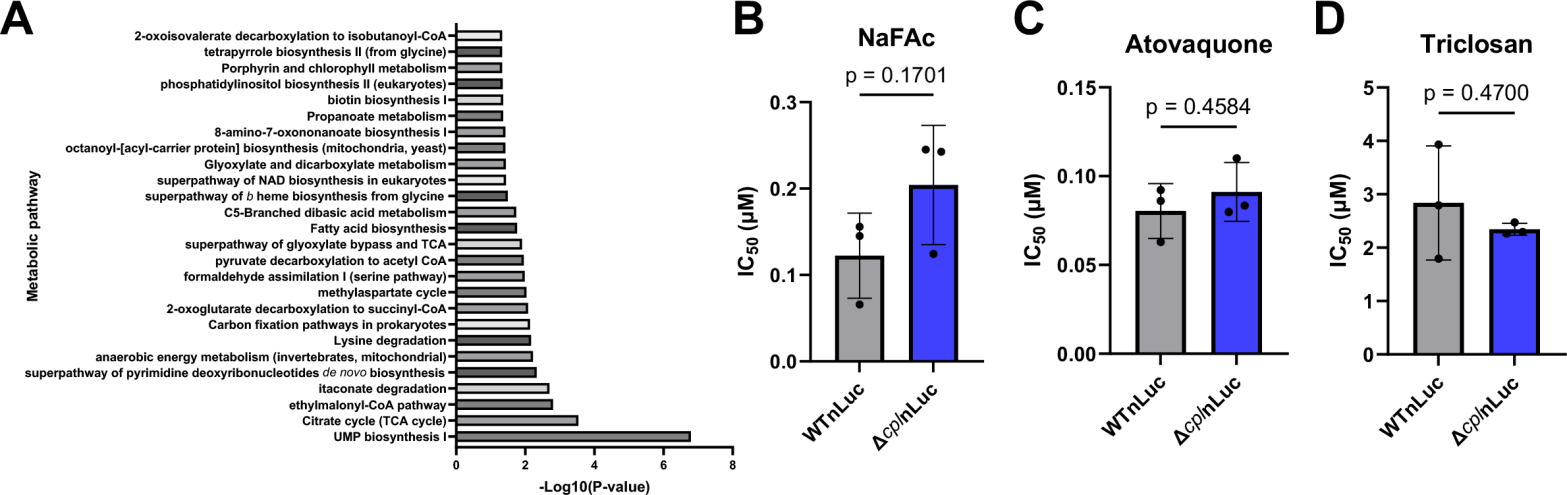
Ingestion mutants rely on several metabolic pathways. (A) Pathway enrichment analysis of the significantly more fitness-conferring hits shared between the RHCas9Δ*cpl* and RHCas9Δ*crt* screens showing KEGG and MetaCyc hits from ToxDB using default settings, with duplicate pathways removed and single-gene pathways excluded. (B–D) Measurement of 50% inhibitory concentration (IC_50_) values of TCA-cycle inhibitors for tachyzoite growth. Confluent HFF monolayers in 96-well plates were infected in triplicate with WTnLuc or Δ*cpl*nLuc parasites in different concentrations of the TCA-cycle inhibitor sodium fluoroacetate (NaFAc) (B), the electron transport chain inhibitor atovaquone (C), or the FA biosynthesis inhibitor triclosan (D). After 4 days at 37°C in 5% CO_2_, parasite growth was measured by luciferase activity. Luminescence values were normalized to growth in vehicle (DMSO), and the IC_50_ was calculated for parasite growth in each drug. The dots are the mean IC_50_ values, with the error bars denoting the SDs from three biological replicates. The Mann-Whitney test was used.

The Δ*cpl* and Δ*crt* samples had the largest overlap of shared hits. Interestingly, all six genes required for pyrimidine biosynthesis (TGGT1_210790, TGGT1_259660, TGGT1_259690, TGGT1_291640, TGGT1_293610, and TGGT1_308580) were among the overlapping genes. Also, four overlapping genes are involved in fatty acid (FA) biosynthesis (TGGT1_217740, TGGT1_221320, TGGT1_225990, and TGGT1_251930), eight in the tricarboxylic acid (TCA) cycle (TGGT1_215280, TGGT1_219550, TGGT1_244200, TGGT1_290600, TGGT1_305980, TGGT1_309752, TGGT1_313140, and TGGT1_314400), and five in lysine degradation (TGGT1_219550, TGGT1_236570, TGGT1_244200, TGGT1_301120, and TGGT1_305980) (Fig. 2A; Table S2).

The gene pairs between the Δ*gra14* mutant and the Δ*cpl* and Δ*crt* mutants did not show significant impacts on metabolic pathways, with the pathway analysis returning only one to two genes per pathway (Table S2). These data suggest that the Δ*cpl* and Δ*crt* mutants might be more reliant on their own biosynthetic pathways due to decreased turnover of material in the PLVAC. In contrast, reduced uptake through the ingestion pathway in Δ*gra14* parasites does not appear to increase the parasites’ reliance on biosynthetic pathways.

To determine whether PLVAC disruption increased reliance on metabolic processes, we tested the ability of RHΔ*ku80*Δ*hxg*nLuc:*hxg* (WTnLuc) and RHΔ*ku80*Δ*hxg*nLuc:*hxg*Δ*cpl:dhfr* (Δ*cpl*nLuc) parasites (34) to grow in the presence of drugs targeting these pathways. Nano luciferase expression allowed us to measure parasite growth via luminescence. We hypothesized that increased reliance on biosynthetic pathways would heighten sensitivity to their disruption. However, WTnLuc and Δ*cpl*nLuc parasites did not have significantly different 50% inhibitory concentration (IC_50_) values when grown in the presence of sodium acetate, atovaquone, or triclosan, which target the TCA cycle, the electron transport chain, and FA biosynthesis, respectively (Fig. 2B through D). While Δ*cpl* was not more susceptible to these drugs, it remains unclear if there was an effect on the metabolomic profile of these parasites.

Our metabolic pathway analysis motivated us to conduct bulk metabolomics on the ingestion mutants to determine the direct impact of the ingestion pathway loss on the resources available to the parasite. We harvested WT, Δ*gra14*, Δ*cpl*, and Δ*crt* mutants after 44 h of growth in confluent human foreskin fibroblast (HFF) monolayers. Additionally, we included two WT samples treated with morpholinurea-leucine-homophenylalanine-vinylsulfone-phenyl (LHVS), a chemical inhibitor of CPL, and dimethyl sulfoxide (DMSO) as a vehicle control, providing comparative points for our WT and Δ*cpl* samples.

Principal component analysis of detected metabolites showed that WT and WT-DMSO clustered together, while Δ*gra14*, Δ*cpl*, and Δ*crt* and four out of six WT-LHVS samples clustered together and away from the WT samples, indicating conserved changes in these parasites (Fig. S4A). We calculated the log2 fold change of metabolites in the ingestion mutants vs WT and LHVS vs DMSO condition (Table S2, log2FC Meta; Fig. S4B). However, this left us with many metabolites that did not slot into pathways detected in our pathway analysis, so we restricted further analysis to the central carbon metabolism, pentose phosphate pathway (PPP), the TCA cycle, energy molecules, nucleosides, and amino acids. We noticed that most metabolites in the Δ*cpl* and LHVS samples followed similar trends (Fig. S4C) but were not identical, possibly due to LHVS inhibiting both parasite CPL and host cathepsin L, thus impacting the host resources available for parasitic scavenging.

**Fig 3:**
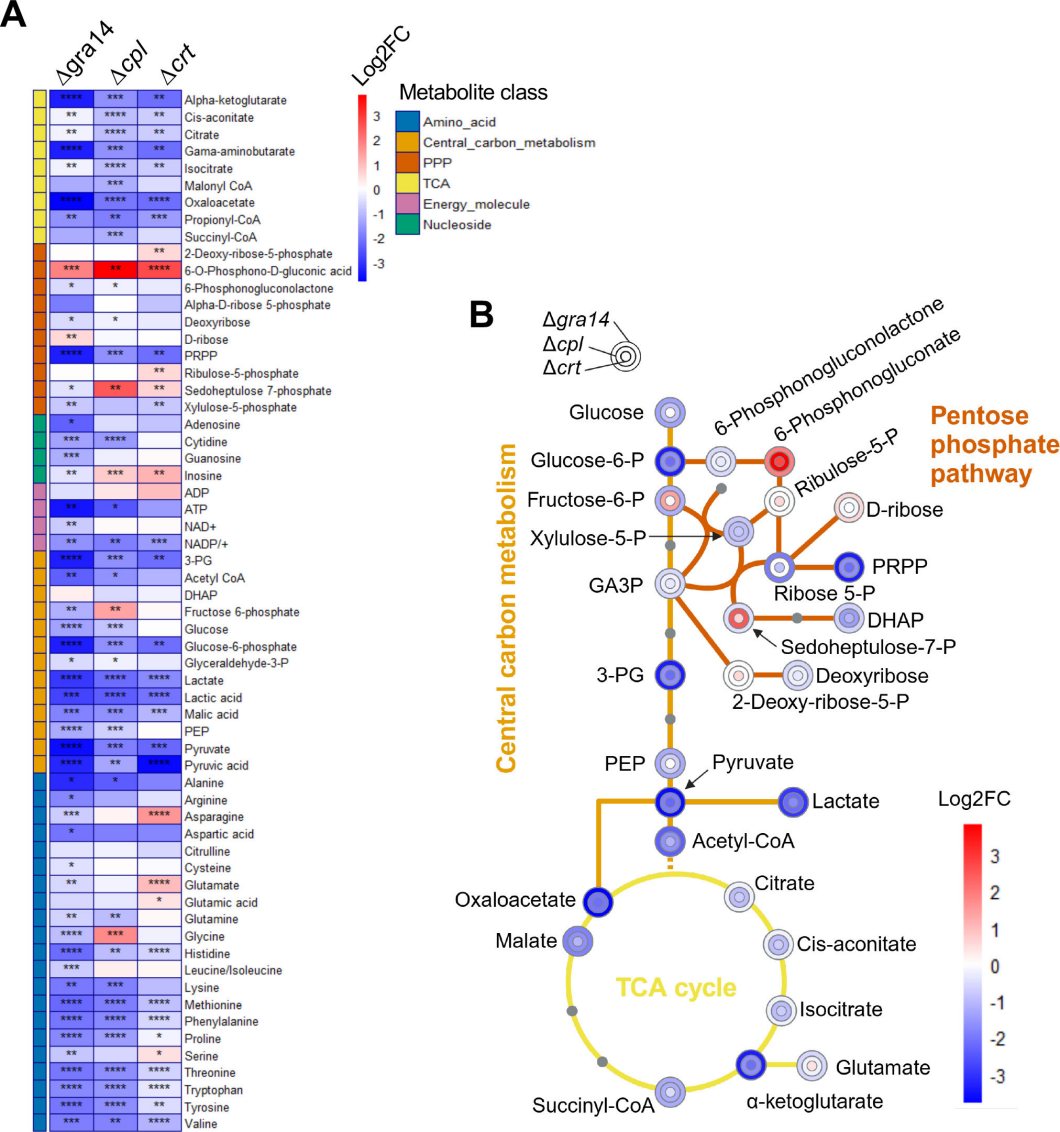
Metabolic analysis of ingestion mutants shows a decrease in amino acids and glycolic end products. (A) heat map of select metabolites from bulk metabolomics data for the ingestion mutants, RHCas9Δ*gra14,* RHCas9Δ*cpl*, and RHCas9Δ*crt*. Parasites infected HFFs for 44 h before the monolayer was harvested. Parasites were freed from host cells and washed to remove residual media, then subjected to liquid chromatography-mass spectrometry. Each metabolite was normalized to the RHCas9WT condition, then the log2FC was calculated. *P < 0.5, **P < 0.01, ***P < 0.001, ****P < 0.0001; *t*-test. *n* = 6 biological replicates. (B) Metabolic pathway schematic for RHCas9Δ*gra14*, RHCas9Δ*cpl*, and RHCas9Δ*crt* depicting the central carbon metabolism (glycolysis and fermentation) in orange feeding into the citric acid cycle in yellow or the pentose phosphate pathway in dark orange. The color of the dot is the log2FC that metabolite experienced in the mutant parasite. Gray dots are metabolites that were not looked at in our analysis.

Continuing the analysis with only the WT, Δ*gra14*, Δ*cpl*, and Δ*crt* samples, we observed several trends from our bulk metabolomics data. Intermediates and end products of glycolysis were reduced in the ingestion mutants (Fig. 3A and B). Specifically, pyruvate was significantly decreased in all three mutants, and glucose was significantly reduced in Δ*gra14* and Δ*cpl* mutants. Within the PPP, 6-O-phosphono-D-gluconic acid was increased in all the ingestion mutants. Since ribose is a main product of the PPP, these findings suggest the need for increased pyrimidine biosynthesis due to disrupted nucleoside salvage. This was further supported by significantly lower levels of guanosine, adenosine, and cytidine in Δ*gra14* parasites (Fig. 3A). Precisely how the ingestion pathway is connected to nucleoside salvage remains to be determined.

The ingestion mutants exhibited markedly reduced levels of most amino acids, with Δ*gra14* mutants showing significantly reduced levels of all amino acids except alanine and valine, which still trended lower (Fig. 3A). The Δ*cpl* mutant had higher levels of glycine, while leucine and isoleucine trended upward. While the Δ*crt* mutant showed higher levels of glutamic acid, serine, and three other amino acids trended upward. These data support a role for the ingestion pathway in scavenging host cytosolic proteins. Despite fewer amino acids in the mutants, there is little to no growth defect compared to WT parasites, suggesting the parasite has an excess pool of amino acids and that it can acquire sufficient amino acids through other means such as amino acid transporters.

To assess if the ingestion pathway significantly contributes to amino acid acquisition under nutrient-limiting conditions, we grew the mutants in single amino acid-depleted media. Using the nano-luciferase strain, we generated Δ*gra14*nLuc and Δ*crt*nLuc strains by disrupting the gene locus and replacing the coding sequence with a *dhfr* resistance cassette, like what was done for the Δ*cpl*nLuc strain (34) (Fig. S5). Starving HFFs for 24 h before parasite infection ensured reduced internal amino acid stores. We focused on tryptophan and phenylalanine, which were significantly reduced in all three mutants, and included tyrosine and arginine due to tyrosine being the other aromatic amino acid, host arginine levels being controlled by host cells, and *Toxoplasma* being auxotrophic for both amino acids (13, 35, 36).

**Fig 4:**
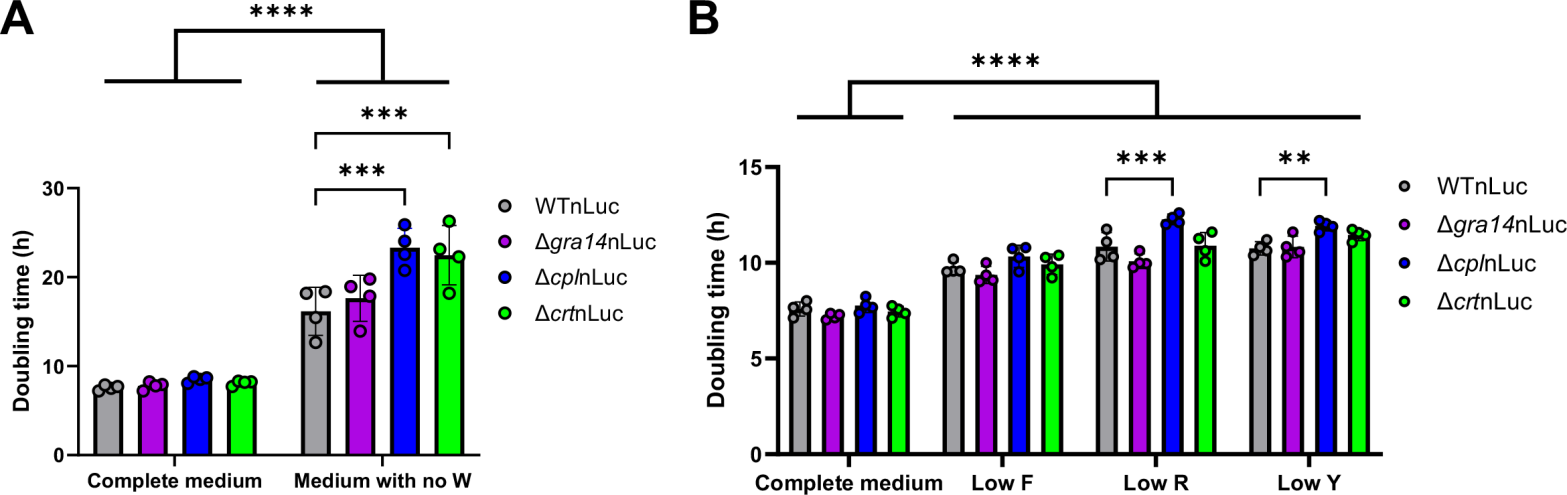
Nutrient limiting conditions inhibit ingestion mutant replication. (A and B) HFF monolayers grew to confluency over 1 week in 96-well plates. Twenty-four hours prior to infection, D10 was replaced with complete D1 or D1 without tryptophan (A) or with 6.5 µM phenylalanine, 8.6 µM arginine, or 3.4 µM tyrosine (B). WTnLuc, Δ*gra14*nLuc, Δ*cpl*nLuc, or Δ*crt*nLuc parasites infected monolayers in triplicate per condition. Luciferin was added at 4, 24, 48, and 72 h post-infection to measure parasite growth. Samples were normalized to the 4 h post-infection reading then log2-transformed. The slope of the linear regression is the doubling time of the parasites. The dots represent mean doubling times, with the error bars denoting the SDs. **P < 0.01, ***P < 0.001, ****P < 0.0001; two-way ANOVA with Tukey’s multiple comparison. *n* = 4 biological replicates.

We assayed parasite growth in complete (D1) and tryptophan-free (D1-W) media to determine doubling time over 3 days. There was no difference in doubling time for WTnLuc, Δ*gra14*nLuc, Δ*cpl*nLuc, and Δ*crt*nLuc parasites in complete media. However, in tryptophan-free media, Δ*cpl*nLuc and Δ*crt*nLuc mutants grew significantly slower than WTnLuc parasites (Fig. 4A). Parasites did not grow in phenylalanine, arginine, or tyrosine-free media, so we tested diminishing concentrations of these amino acids on WTnLuc parasites to identify minimal growth conditions (Fig. S6). Testing parasites in 6.5 µM F, 8.6 µM R, or 3.4 µM Y showed smaller shifts in overall doubling time, yet Δ*cpl*nLuc still grew significantly slower in arginine- and tyrosine-limiting conditions (Fig. 4B). Additionally, all nutrient limited growth conditions grew slower than the complete media (Fig. 4A and B). Overall, these data suggest that *Toxoplasma* may rely on material turnover in the PLVAC during nutrient-limited situations, facing difficulties in resource scavenging.

## DISCUSSION

Our results provide insights into the adaptive mechanisms employed by *Toxoplasma* in the absence of a functional ingestion pathway. Despite modest bottlenecks in our CRISPR screens, comparison of the WT screen done herein to the original screen by the Lourido Lab (29) showed a strong correlation. Furthermore, we noted statistically significant overlaps in hits shared by pairwise analysis of Δ*gra14*, Δ*cpl*, and Δ*crt*. The overlap between Δ*cpl* and Δ*crt* was particularly strong and involved pathways for pyrimidine biosynthesis, FA biosynthesis, the TCA cycle, and lysine degradation. This suggests a pivotal compensatory role for these biosynthetic pathways when the PLVAC is disrupted. However, we were unable to confirm increased reliance on the pathways identified by metabolic pathway analysis via our drug sensitivity assay. Our bulk metabolomic profiles of the ingestion mutants complemented our pathway analysis findings, revealing a pronounced deficiency in glycolytic intermediates and certain amino acids, suggesting a marked alteration of these biosynthetic pathways when the ingestion pathway is compromised.

Our detailed metabolomic analysis supports the hypothesis that loss of the ingestion pathway significantly impacts amino acid acquisition. Mutant strains exhibited a global decrease in most amino acids, indicative of disrupted amino acid salvage from the host cytosol. Notably, the Δ*gra14* mutant displayed markedly reduced levels of all amino acids except alanine and valine, while the Δ*cpl* and Δ*crt* mutants showed a slightly more complex but similar amino acid profile disruption. This supports the notion that the ingestion pathway functions in scavenging key nutrients, particularly amino acids, from the host.

We explored this hypothesis by growing the mutants in single amino acid-depleted media. The lack of a differential growth defect in Δ*gra14* vs WT parasites during amino acid deprivation, despite amino acid levels being most disrupted in this mutant, suggests that there is sufficient access to the resources for growth. This may be due to redundant GRA proteins still acquiring sufficient nutrients from the host in the form of other macromolecule uptake systems or diffusion of soluble nutrients across the nutrient pore. Conversely, if the PLVAC is a hub of exogenous and endogenous amino acid turnover, even with reduced amino acid uptake in Δ*gra14* parasites, sufficient resources may be recycled for parasite growth. This is consistent with our findings that, under nutrient limitation, PLVAC mutants displayed growth impairments, especially in conditions deficient in tryptophan, arginine, and tyrosine. Our findings suggest that *Toxoplasma* uses the PLVAC as a site of protein turnover for amino acids during host nutrient deprivation, such as during host infection-induced amino acid starvation.

Interestingly, in our Δ*cpl* CRISPR screens, two of the four known nutrient pore genes, GRA17 and GRA72, were detected as more fitness conferring (Fig. 1D). Supportive of this interaction, the GRA17 synthetic lethal screen also observed a negative shift in the phenotype score of CPL, though it was not interrogated for a synthetic lethal phenotype (10). This raises an interesting hypothesis that when protein turnover is impaired by loss of CPL, the parasite might rely more heavily on soluble nutrients as ready-to-use material. Speculatively, this could indicate that high affinity transporters on the parasite plasma membrane for the resources being obtained by GRA17 may be detected in our Δ*cpl* CRISPR screen, which could offer interesting insights into the critical material the PLVAC is turning over.

Our study provides valuable insights into the adaptive resilience and metabolic flexibility of *Toxoplasma*. This is highlighted in the metabolomic data and pathway analysis that showed a decrease in nucleosides and an increased reliance on pyrimidine biosynthesis in these mutants. These data suggest that the ingestion pathway may also scavenge host cytosolic nucleosides, possibly in the form of host rRNA, mRNA, and tRNA. Recently, a preprint study localized TgENT1, an equilibrative nucleoside transporter, to the PLVAC (37). This transporter, which is essential to the parasite, could play a role in liberating host nucleosides that were acquired via the ingestion pathway.

Among the limitations of this study, we were unable to directly validate any of the overlapping hits as being synthetically lethal. Of the 30 genes that were consistently more fitness conferring in all three ingestion-deficient mutants, only 9 of the hits had WT phenotype scores that were indicative that they might be dispensable in WT parasites. However, upon looking at data from previous screens, we can see that five of these genes, TGGT1_262640, TGGT1_221320, TGGT1_286530, TGGT1_288880, and TGGT1_249270, have phenotype scores of <−2, which indicates another screen found them fitness conferring in WT parasites (29). This highlights the importance of recognizing a degree of uncertainty with phenotype scores. Also, we attempted to validate the hits by measuring viability (plaquing efficiency) and overall progression through the lytic cycle (plaque size). These assays do not measure fitness in the same way as the CRISPR screen, which is effectively a competition assay. Alternative ways of validating hits for synthetic lethal screens, including pairwise competition assays, should be considered.

Our findings underscore the reliance of *Toxoplasma* on a multiplicity of adaptive pathways to compensate for the loss of nutrients. This flexibility ensures that the parasite can thrive under various constraints, highlighting its evolutionary success as an intracellular pathogen.

## MATERIALS AND METHODS

### Host cells and cell culture

HFFs (American Type Culture Collection) were maintained in Dulbecco’s modified Eagle’s medium (DMEM) with 4.5 g/L glucose supplemented with 2 mM L-glutamine, 10% Cosmic calf serum, 20 mM HEPES, and 5 µg/mL pen/strep (D10) at 37°C and 5% CO_2_ unless otherwise stated. *Toxoplasma gondii* strains were passaged in the above HFFs.

### Parasite strain generation

A list of all parasite strains, primers, and plasmids can be found in Tables S4 to S6, respectively. To generate RHCas9*Δgra14*, RHCas9 parasites were transfected with 50 µg of circular pU6-DHFR-GRA14gRNA1, which targets the first exon of the gene. To prevent integration of the resistance marker, the plasmid was not linearized. After 24 h, media were changed. Once the HFF monolayer was lysed, parasites were syringed, filtered, and diluted into 96-well plates to isolate single clones. Clones were grown for 1 week and expanded to 24-well plates for DNA extraction. DNA was extracted using a Qiagen DNeasy Blood and Tissue kit and subjected to PCR by the Q5 polymerase with primers, P1 and P2, which flanked the gRNA cut site. Purified fragments were sent for Sanger sequencing to identify INDEL mutations. The same procedure was used to generate RHCas9Δ*crt* using pU6-DHFR-CRTgRNA 1 and primers 3 and 4. RHCas9Δ*cpl* was generated similarly, but pCas9-GFP-Ble-CPLgRNA1 was used to allow for sorting of GFP +parasites. Twenty-four hours after transfection, the monolayer was scraped, syringed, and filtered. Parasites were resuspended in phosphate-buffered saline (PBS) + 5% FBS and sorted by flow cytometry. GFP+ parasites were used to infect HFFs. Once this monolayer was lysed, the procedure above was followed, and primers 5 and 6 were used to verify the INDEL. Protein disruption was validated by western blotting using antibodies specific to these proteins.

To generate RHΔ*ku80nLuc*Δ*gra14*, RHΔ*ku80*nLuc parasites were transfected with 50 µg of pCas9-GFP-Ble-GRA14gRNA1 and 10 µg of repair template that had 40 bp of homology to the 5′ and 3′ of the genomic DNA, flanking a DHFR resistance cassette. Twenty-four hours post-transfection, the medium was changed, and 3 µM pyrimethamine was added for selection. Once the population was lysed, the monolayer was scraped, syringed, filtered, and cloned into 96-well plates. After 1 week, the Phire Tissue Direct PCR kit was used to test for integration of the DHFR resistance cassette using primers P9 and P10 for 5′ integration. This was also validated at the protein level by western blotting. A similar approach was used to generate RHΔ*ku80nLuc*Δ*crt*. However, 2 sgRNAs, pCas9-GFP-Ble-CRTgRNA1 and pCas9-GFP-Ble-CRTgRNA2, were used as *CRT* is a multi-exon gene. Primers P3 and P10 were used to test for 5′ integration.

### Ingestion assay

Inducible mCherry HeLa cells and harvesting steps were used from a previous study (19). After harvest, parasites were fixed with 4% methanol-free formaldehyde and imaged on a Zeiss Axiovert Observer fluorescence microscope. Samples were blinded, and mCherry+ parasites were counted. Data were graphed and analyzed in Prism.

### Immunoblot

A list of all antibodies is in Table S7. Parasite lysates were harvested using radioimmunoprecipitation assay(RIPA) buffer solution (Thermo Scientific, 89900) with EDTA-free miniComplete protease inhibitor (Roche, 11836153001). PVDF membranes were activated with methanol for protein transfer, then blocked with 5% milk in PBS-Tween. The primary antibody was incubated O/N at 4°C or for 1 h at room temperature (RT) for loading controls, followed by horseradish peroxidase-conjugated secondary antibodies for 1 h at RT. The signal was developed with 2 min incubation in SuperSignal West Pico PLUS Chemiluminescent or SuperSignal West Femto Maximum sensitivity substrate. The chemiluminescent signal was visualized using the Syngene PXi6 imaging system. Membranes were stripped using 4% trichloroacetic acid then blocked and re-probed as needed.

### CRISPR screens

Genomewide screens used an sgRNA library (CrLib) containing 10 sgRNAs per gene for 8,156 genes in *Toxoplasma*, following an established protocol (28) with few changes. The CrLib was linearized using AseI, split into 12 cuvettes for transfection (50 µg of linearized plasmid with 5 × 10^7^ parasites each), and used to infect 12 D150 dishes of HFFs. Twenty-four hours post-infection, media were changed to D10 with 40 µM chloramphenicol, 3 µM pyrimethamine, and 10 μg/mL DNase1. To maintain parasite diversity, 1.5 × 10^8^ total parasites were passaged every 48–72 h, based on monolayer integrity, for four passages. After the final passage, 1 × 10^8^ parasites were pelleted, and genomic DNA was extracted using the Qiagen DNeasy Blood and Tissue kit. The sgRNAs were amplified from genomic DNA using the primers listed in Table S4 and gel purified for Illumina NovaSeq sequencing. Three sequential screens were performed for each mutant and the WT parasites.

### Data analysis

A custom KNIME workflow trimmed the sequencing reads to begin with the sgRNA sequence, making them compatible with the Countess R script (https://github.com/LouridoLab/CRISPR_Analysis), detailed in the protocol paper (28). The raw counts can be found in Table S1. The phenotype score was the log2 fold change for the five most abundant gRNAs for each gene. To determine which genes underwent a change in phenotype score, we compared the average mutant score to the average WT score. A shift was significant if it was greater than 2 SDs above the average shift of the 497 sexual genes (32) experienced in the mutant compared to the WT. Additionally, one-sided Wilcoxon signed-rank test was used to determine the probability of each gene having a significant shift compared to the shift in the 497 sexual genes (FDR < 0.01). Genes significant by both methods were classified as hits. We assessed significant overlaps between screens by using hypergeometric overlap testing (http://nemates.org/MA/progs/overlap_stats.html).

### Gene enrichment analysis

Genes with significant shifts were subjected to gene enrichment analysis using the metabolic pathway enrichment analysis from ToxoDB, using default settings. These results can be found in Table S2.

### Validation of screens

Thirty genes were fitness conferring genes in all three mutants. Of these, six genes had fitness scores of >−2.5 in the WT, suggesting they were dispensable. To validate synthetic lethality, these genes were disrupted in the RHCas9WT, RHCas9Δ*gra14*, and RHCas9Δ*cpl* parasites, and the fitness of the resulting single or double mutants was determined. pU6-DHFR-SAG1 and pU6-DHFR-CDPK1 sgRNA served as negative and positive lethality controls, respectively. For knockouts, 5 × 10^7^ parasites were transfected with 50 µg of linearized plasmid and plated in six-well plates in duplicate. Ethanol treated wells were seeded with 100 and 1,000 tachyzoites. Pyrimethamine (1.5 µM) treated wells were seeded with 1,000 or 10,000 tachyzoites. Ethanol-treated plates grew for 8 days; pyrimethamine-treated plates grew for 10 days. On the final day of growth, supernatant from the pyrimethamine-selected RHCas9WT plates was collected for validation of gene disruption (detailed below). For the plaque assay, monolayers were washed with PBS, fixed with 4% methanol-free formaldehyde, and stained with 2% crystal violet in 20% ethanol. Wells were imaged on a Nikon Ti-2E microscope at ×4 magnification and stitched together using NIS-Elements. Two wells were analyzed per condition. Plaques were detected and quantified using the plaque assay module in SpaCR (v. 0.3.62, https://github.com/EinarOlafsson/spacr and https://pypi.org/project/spacr/). First, plaques were manually annotated using the SpaCR module spacr.make_masks on 100 stitched whole-well images. The Cellpose cyto model (38) was fine-tuned (39) on 90 images with the spacr.train module (Table S9). Ten images were reserved for testing. The plaque counts and sizes were computed by SpaCR. The data were imported to R, where outlier analysis was conducted, and average plaque size and count for each condition were calculated and normalized to the EtOH and Δ*sag1* controls. The data were exported to PRISM for graphing and statistical analysis.

To confirm successful gene knockout using this strategy, the supernatant from plaques was collected and plated for single clones in 96-well plates. After 7 days, the Phire Tissue Direct PCR kit was used to probe for disruption of the gene through the integration of the sgRNA fragment at the targeted gene locus with genomic and sgRNA primer pairs (P57–P70 and P55 and P56). Clones negative for a genomic-sgRNA PCR fragment were subjected to PCR with genomic primer pairs that flanked the cut site. That PCR fragment was cleaned up and sent for Sanger sequencing to identify INDELs.

### Luciferase growth assay

To determine if Δ*cpl*nLuc parasites were more susceptible to drugs that targeted the metabolic pathways that were more fitness conferring in the Δ*cpl* and Δ*crt* mutants, we employed a growth assay. Briefly, 96-well plates were seeded with HFFs 1 week prior to infection. On the day of infection, WTnLuc and Δ*cpl*nLuc parasites were harvested by scraping, syringing, filtering, and centrifugation at 1,200 × *g* for 10 min, then washed and re-suspended in HBSS. Triplicate wells for each strain were infected with 2,000 tachyzoites per well. Parasites invaded for 4 h before the wells were washed with PBS, and fresh media were added containing either DMSO or three-step serial dilutions of sodium fluoroacetate from 5.4 µM, atovaquone from 5.4 µM, or triclosan from 17 µM. After 4 days, media were aspirated from the wells, and monolayers were lysed for 10 min in 50% 1× PBS 50% 2× lysis buffer [100 mM 2-(N-morpholino)ethanesulfonic acid (MES), 1 mM 1,2-cyclohexylenedinitrilotetraacetic acid (CDTA), 0.5% Tergitol, 0.05% Antifoam 204, 150 mM KCl, 1 mM dithiothreitol (DTT), and 35 mM thiourea] with 12.5 µM h-coelenterazine (NanoLight Technology, 301-10) as the luciferase substrate. Luminescence was measured on a Syngene plate reader using the following settings: integration time: 1 s, optics: top, gain: 135, delay: 100 ms, and read height: 4.5 mm. Data were normalized to the DMSO controls; growth curves were plotted; and the IC_50_ was calculated in Prism.

To assess growth under nutrient-limited conditions, we adapted a luciferase growth assay similar to reference 34. Briefly, 96-well plates were seeded 1 week prior to infection in D10. Twenty-four hours prior to infection, the D10 was replaced with amino acid-free DMEM (US Biological, D9800-13) supplemented with 1% dialyzed fetal bovine serum, 20 mM HEPES, and 5 µg/mL pen/strep and further supplemented as described in Table S8 to make D1 complete, D1 -tryptophan, D1 -phenylalanine, D1 -arginine, and D1 -tyrosine. The media were brought to pH 7.4–7.6 before filter sterilization. D1 complete was mixed with an amino acid-free media to make the necessary two-step concentrations. To determine inhibitory concentrations for phenylalanine, arginine, and tyrosine, triplicate wells were infected as above. After invasion and washing, the two-step serial dilution of D1 was re-applied to the wells. The first reading for normalization was gathered at this time. Reading of parasite growth was accomplished as above. For inhibitory concentrations of F, R, and Y samples were assayed after 4 days of growth and normalized to the 4 h post-infection reading and plotted as a percentage of the D1 complete growth. A non-linear curve was fit to the graphs in PRISM to estimate the EC_90_.

For growth assays in nutrient-limited media, 96-well plates were seeded with HFFs and grown for 1-week in D10. Twenty-four hours prior to infection, D10 was swapped for amino acid limited D1. WT*nLuc*, Δ*gra14nLuc*, Δ*cplnLuc*, and Δ*crtnLuc* parasites were harvested as above. Triplicate wells for each strain and time point captured were infected as above. Readings were taken at 4, 24, 48, and 72 h post-infection and were normalized to the 4 h post-infection readings for each sample before the fold change was calculated. Prism was used to calculate the doubling time of the parasites.

### Metabolomics

HFF monolayers were infected with an equal number of RHCas9, RHCas9Δ*gra14*, or RHCas9Δ*cpl*, and RHCas9Δ*crt* parasites. After 2 h of invasion, the monolayer was washed with DMEM, and infection proceeded for 18 h in D1 complete. Samples were either treated with 1 µm LHVS for 24 h or DMSO as the vehicle control before parasites were syringe lysed and harvested for metabolomic analysis using liquid chromatography-tandem mass spectrometry. Untargeted metabolomics was conducted as previously published (40). Data were analyzed in R to identify outliers, calculate the log2 fold change, and determine the significance of the metabolites between the WT and ingestion mutants, as well as the DMSO and LHVS conditions.

## SUPPLEMENTAL MATERIAL

Figure S1 msphere.01011-24-s0001.tif Validation of RHCas9 knockouts.

Figure S2 msphere.01011-24-s0002.tif Quality control analysis of genome-wide CRISPR screens.

Figure S3 msphere.01011-24-s0003.tif Analysis of plaque efficiency and size for screen hits.

Figure S4 msphere.01011-24-s0004.tif Consistent clustering of groups in metabolic data.

Figure S5 msphere.01011-24-s0005.tif Generation of RHΔ*ku80*Δ*gra14*nLuc.

Figure S6 msphere.01011-24-s0006.tif Growth of *Toxoplasma* in phenylalanine-, arginine-, or tyrosine-limiting conditions.

Legends msphere.01011-24-s0007.docx Description of supplemental tables and legends for supplemental figures.

Supplemental tables msphere.01011-24-s0008.xlsx Tables S1 to S9.

ASM does not own the copyrights to Supplemental Material that may be linked to, or accessed through, an article. The authors have granted ASM a non-exclusive, world-wide license to publish the Supplemental Material files. Please contact the corresponding author directly for reuse.
