# Supplementary figures and images for: Metabolic adaptability and nutrient scavenging in Toxoplasma gondii: insights from ingestion pathway-deficient mutants
Source: mSphere. 2025 Apr 2;10(4):e01011-24. doi: 10.1128/msphere.01011-24 (PMC12039266; doi:10.1128/msphere.01011-24)

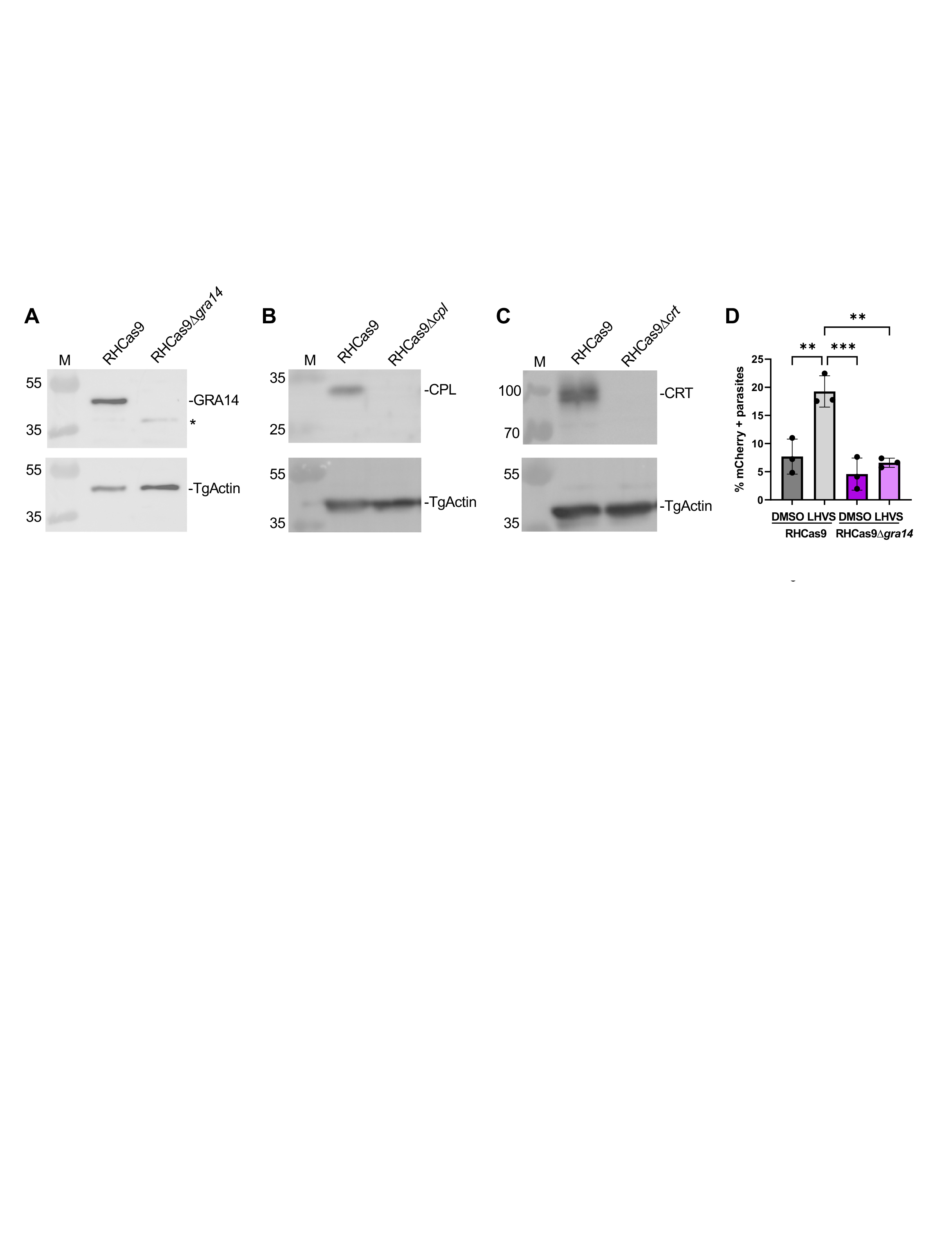

Supplement: Figure S1 — Validation of RHCas9 knockouts. [file msphere.01011-24-s0001.tif]

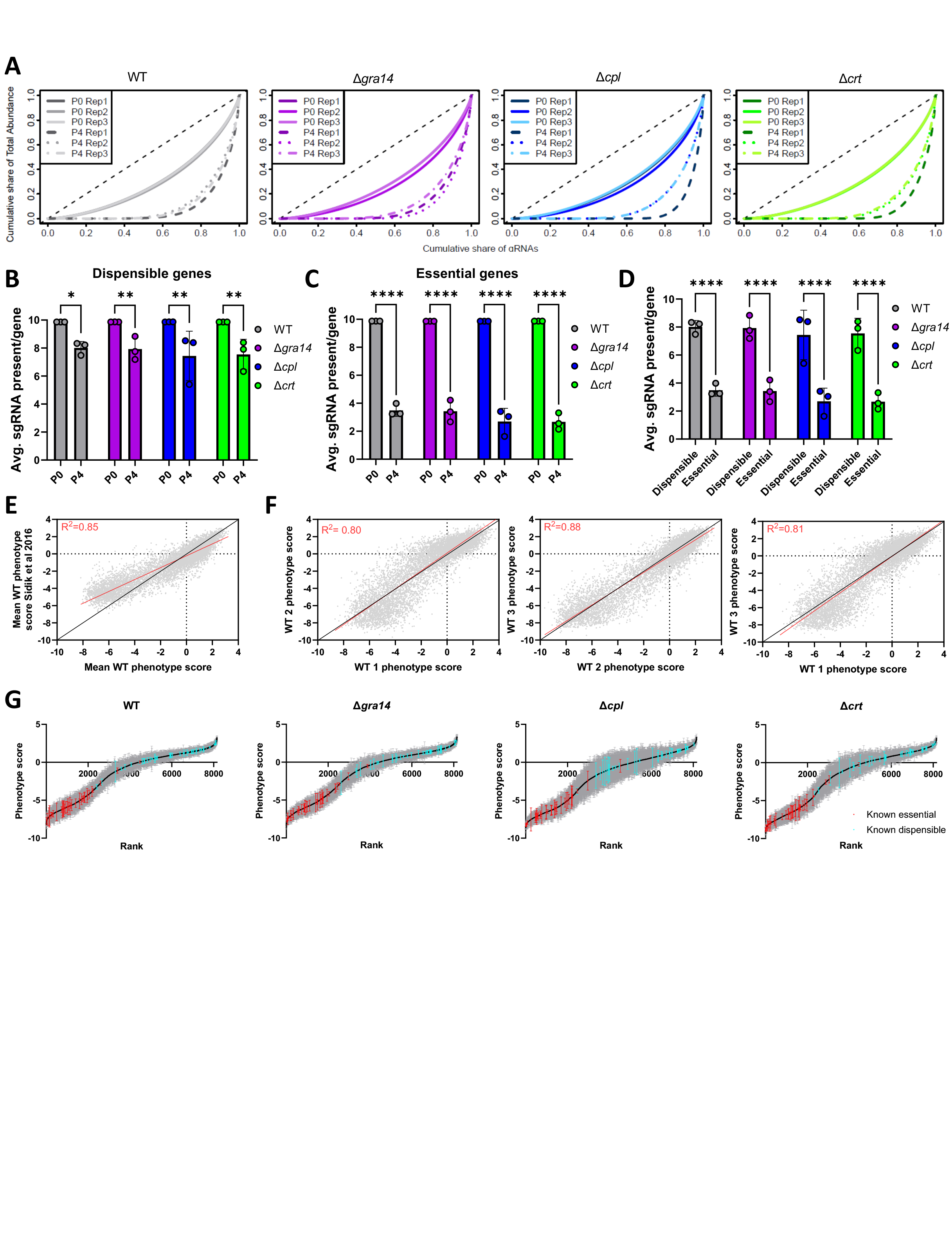

Supplement: Figure S2 — Quality control analysis of genome-wide CRISPR screens. [file msphere.01011-24-s0002.tif]

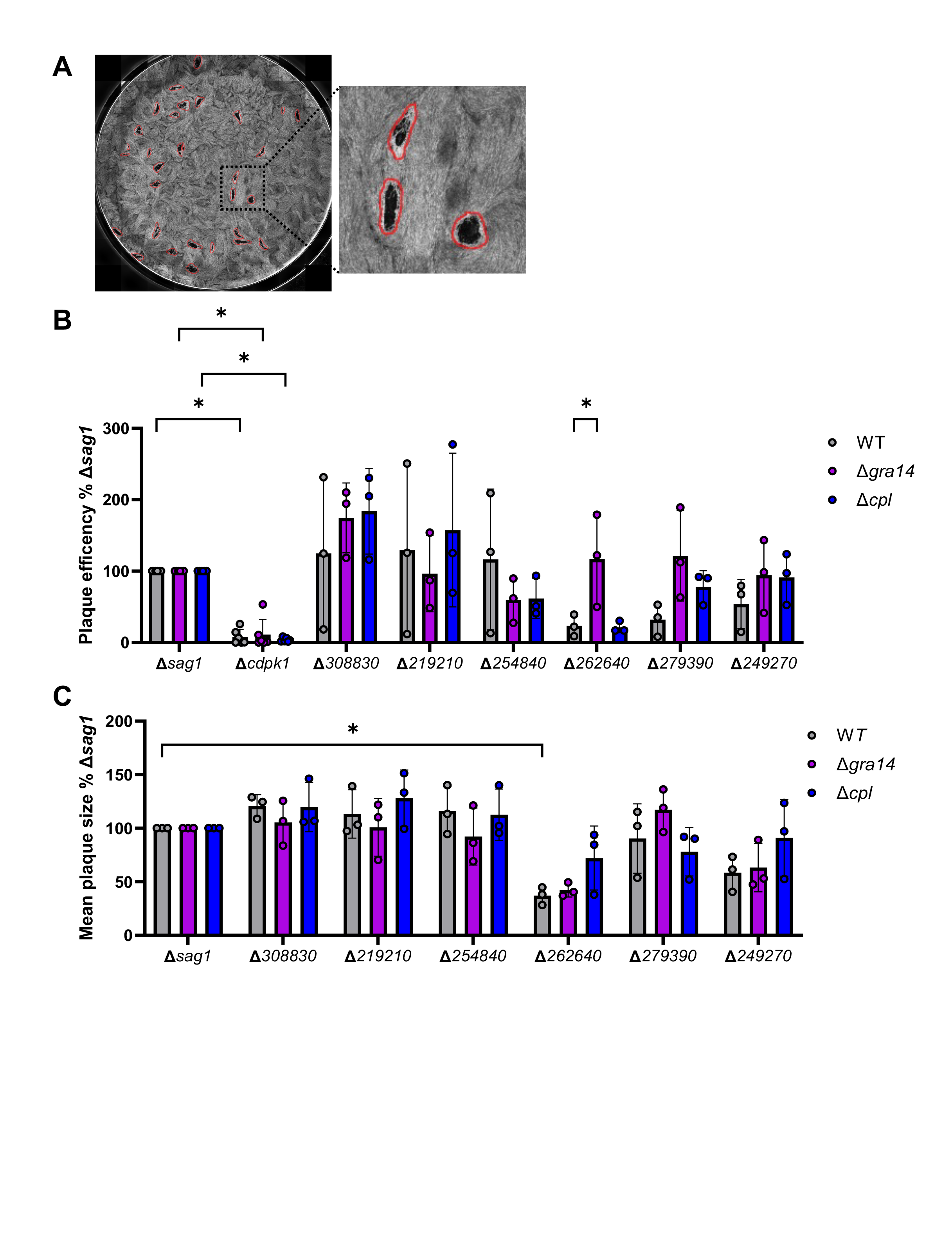

Supplement: Figure S3 — Analysis of plaque efficiency and size for screen hits. [file msphere.01011-24-s0003.tif]

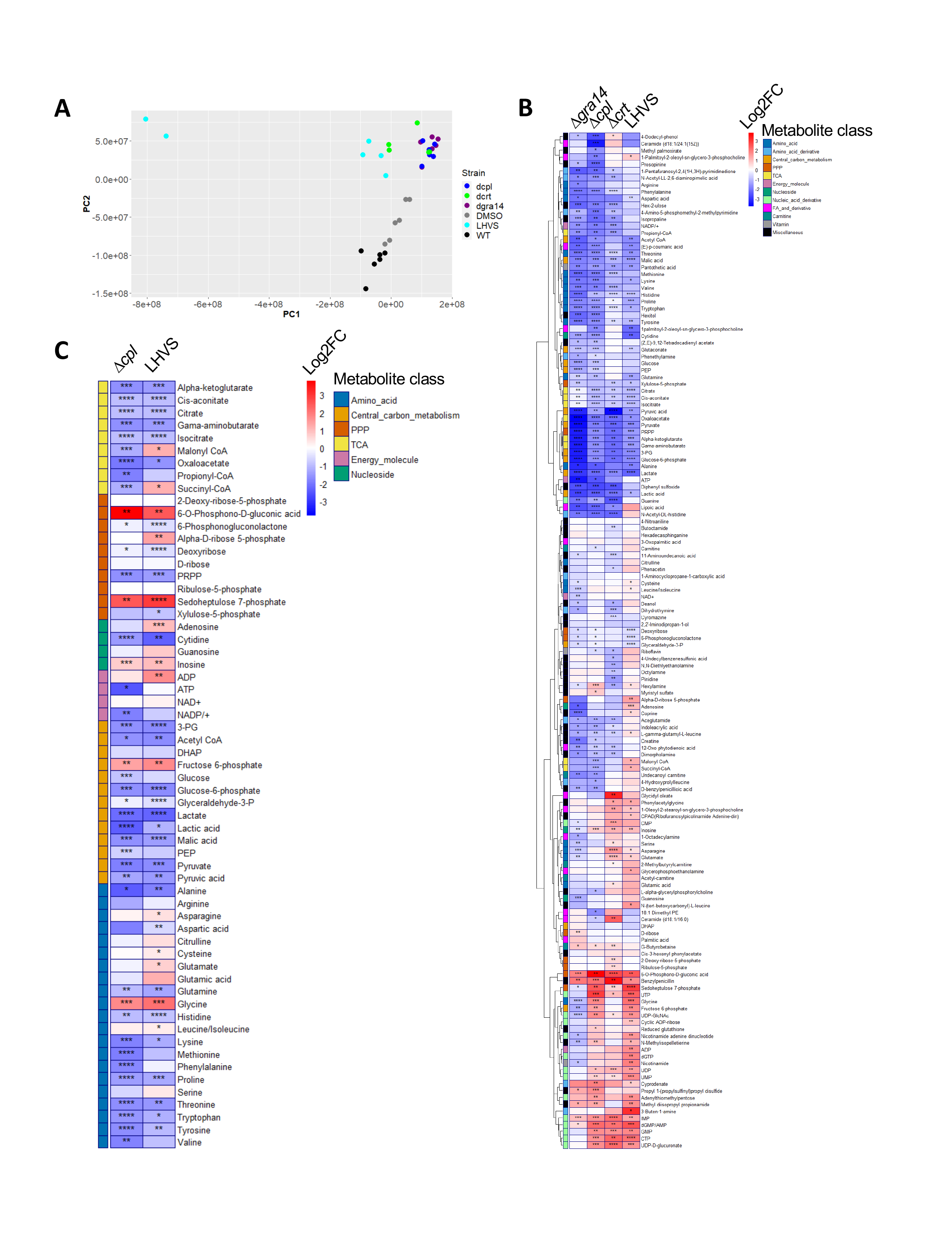

Supplement: Figure S4 — Consistent clustering of groups in metabolic data. [file msphere.01011-24-s0004.tif]

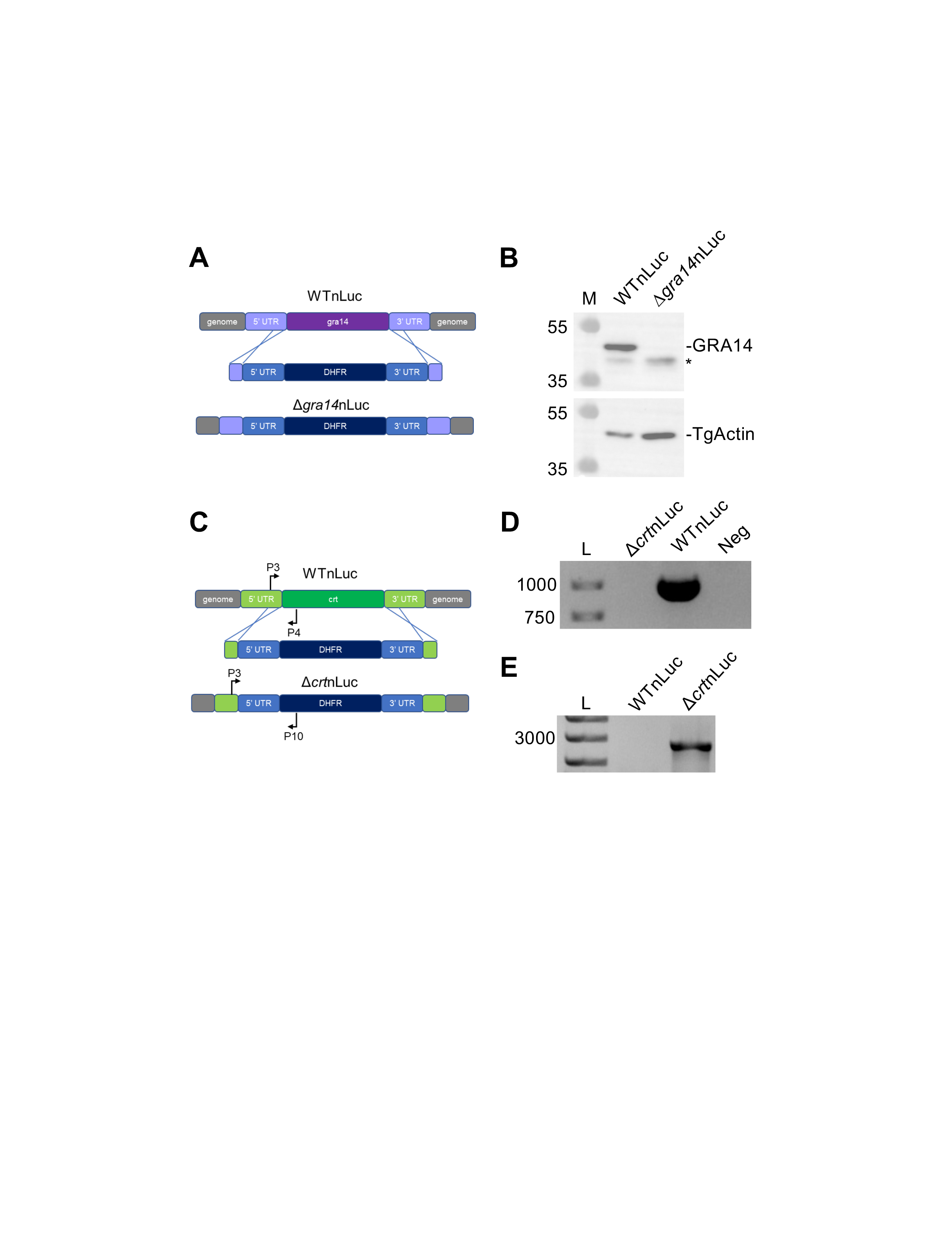

Supplement: Figure S5 — Generation of RHΔku80Δgra14nLuc. [file msphere.01011-24-s0005.tif]

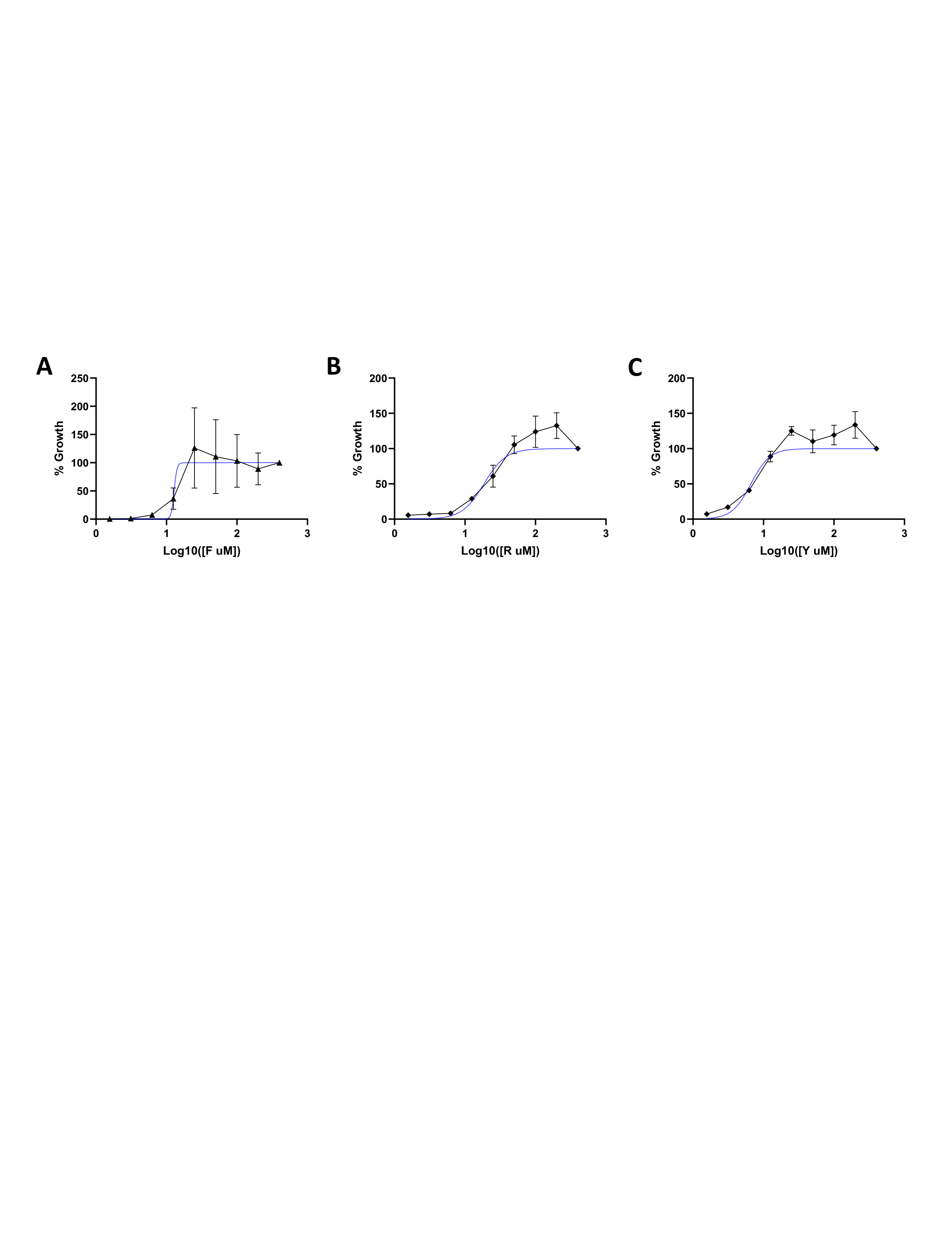

Supplement: Figure S6 — Growth of Toxoplasma in phenylalanine-, arginine-, or tyrosine-limiting conditions. [file msphere.01011-24-s0006.tif]
