# Supplementary material for: Metabolic adaptability and nutrient scavenging in Toxoplasma gondii: insights from ingestion pathway-deficient mutants
Source: mSphere. 2025 Apr 2;10(4):e01011-24. doi: 10.1128/msphere.01011-24 (PMC12039266; doi:10.1128/msphere.01011-24)
Supplement: Legends — Description of supplemental tables and legends for supplemental figures. [file msphere.01011-24-s0007.docx]

**Table S1.** Screen raw count: the raw count of each sgRNA from the initial (P0) and final (P4) library for each sample. Screen data: phenotype score, significance, and gene information from ToxoDB for each gene in the CrLib.

**Table S2.** Pathway enrichment analysis data from ToxoDB for significantly fitness conferring hits from CRISPR screen shared between Δ*gra14*, Δ*cpl*, Δ*crt* and Δ*cpl*, Δ*crt* and Δ*gra14*, Δ*cpl* and Δ*gra14*, Δ*crt*, and the Δ*gra14*, Δ*cpl*, Δ*crt* screens alone.

**Table S3.** Metabolites raw: raw metabolic count for the metabolites detected in the analysis. Log2FC meta: Log2FC and p-values of each metabolite with outliers removed compared to WT for Δ*gra14*, Δ*cpl*, Δ*crt* and DMSO for LHVS samples.

**Table S4.** Strains: strains used in this study

**Table S5:** Primers: primers used in this study

**Table S6:** Plasmids: plasmids used in this study

**Table S7:** Antibodies: antibodies used in this study

**Table S8:** Media: media recipes for amino acid supplementation

**Table S9:** SpaCR settings: settings used during SpaCR training and data analysis.

**Figure S1. Validation of RHCas9 knockouts. (A)** Parasite lysates of RHCas9 and RHCas9Δ*gra14* were probed with RbαGRA14 or MsαTgActin (as a loading control). The astrisk denotes a non-specific band. **(B)** Parasite lysates of RHCas9WT and RHCas9Δ*cpl* were probed with MsαCPL or RbαTgActin. **(C)** Parasite lysates of RHCas9WT and RHCas9Δ*crt* were probed with RbαCRT or MsαTgActin. Molecular weight markers (M) are shown in kDa. **(D)** Quantification of host mCherry ingestion 24 h post-infection by RHCas9 or RHCas9Δ*gra14* parasites treated with DMSO (vehicle) or 1 µM LHVS to inhibit degradation of host mCherry in the PLVAC ** *p*<0.01, *** *p*<0.001. One-way ANOVA with Tukey’s multiple comparison. *n*=3 biological replicates.

**Figure S2. Quality control analysis of genome-wide CRISPR screens. (A)** Lorenz curves of each CRISPR screen. P0 indicates the initial library for transfection and P4 is the final passage population. **(B, C)** The average number of unique sgRNA that were present in the initial and final populations for the dispensable (B) or essential (C) genes. **(D)** The average number of unique sgRNAs that were present per gene for the essential and dispensable genes in the final population of the of each screen. **(E)** Correlation of our RHCas9 phenotype scores mapped to the previously published scores from Sidiki et al., 2016. **(F)** Internal correlation of the phenotype score for each WT-screen replicate against the others. **(G)** Ranked phenotype score graphs with SEM for each screen with known essential (red) and dispensable (cyan) genes marked. **(C)** * *p*<0.05, ** *p*<0.01, **** *p*<0.0001. Two-way ANOVA with Tukey’s multiple comparison. *n*=3 biological replicates.

**Figure S3. Analysis of plaque efficiency and size for screen hits. (A)** Representative image of crystal violet stained well after 4x magnification images were stitched together using Nikon Elements. Masks were created after automated plaque identification was performed using the plaque assay module in spacr, a modified Cellpose cyto model trained to detect plaques in monolayers of cells, on two wells per condition. Masks were counted for plaque count and size was recorded for plaque size analysis. Red outlines trace mask area onto the input image. **(B)** RHCas9WT, RHCas9Δ*gra14,* RHCas9Δ*cpl* parasites were transfected with an sgRNA targeting the indicated gene, and 1×10^3^ or 1×10^4^ parasites were inoculated into a 6-well plate under 3 µM pyrimethamine selection for 10 days. Wells were fixed in 4% formaldehyde and stained with 2% crystal violet before being imaged. Plaque efficiency was calculated as the number of plaques counted versus the number of parasites added to the well, as determined from the plaque counts of non-drug selected wells, then normalized to the Δ*sag1* value for the given strain. **(C)** Plaque size was calculated and normalized to the Δ*sag1* value for the given strain. **B, C** The dots are means with SD as the error bars. * *p*<0.5, two-way ANOVA with Tukey’s multiple comparison, only comparisons between same strain Δ*sag1*:ΔGOI and WT:ingestion mutant ΔGOI are being shown. n=3, except for Δ*sag1* and Δ*cdpk1* in **B** which was *n*=6.

**Figure S4: Consistent clustering of groups in metabolic data. (A)** PCA plot from the RHCas9, RHCas9Δ*gra14,* RHCas9Δ*cpl,* RHCas9Δ*crt*, RHCas9 DMSO and RHCas9 LHVS metabolomics data. *n*=6 **(B)** Heat map of hierarchical clustering of metabolite Log2FC for the ingestion mutants and RHCas9 LHVS normalized to RHCas9 and RHCas9 DMSO, respectively. Parasites infected HFFs for 44 hours, with 24 h of DMSO or LHVS treatment as required, before harvest. Parasites were freed from host cells and washed to remove residual media then subjected to LC-MS. **(C)** Heat map of select metabolites from bulk metabolomics data for RHCas9Δ*cpl,* RHCas9 LHVS. **B, C** The LHVS samples 1 and 4 were removed due to being outliers. Statistical significance was determined by t-test, * *p*<0.5, ** *p*<0.01, *** *p*<0.001, **** *p*<0.0001. RHCas9Δ*gra14,* RHCas9Δ*cpl,* RHCas9Δ*crt n*=6 and RHCas9 LHVS *n*=4.

**Figure S5. Generation of RHΔ*ku80*Δ*gra14*nLuc. (A)** Schematic of replacing the *gra14* locus with DHFR **(B)** Parasite lysates of RHΔ*ku80*nLuc, RHΔ*ku80*Δ*gra14*nLuc with RbαGRA14 or MsαTgActin as a loading control. Molecular weight markers (M) are shown in kDa. The asterisk denotes a nonspecific band. **(C)** Schematic of replacing the *CRT* locus with DHFR **(D)** Genomic DNA of WTnLuc and Δ*crt*nLuc PCR’d with P3 and P4 to look for the native *CRT* locus. **(E)** Genomic DNA of WTnLuc and Δ*crt*nLuc PCR’d with P3 and P10 to assay 5’ integration. **D, E** DNA Ladder (L) is shown in bp.

**Figure S6. Growth of *Toxoplasma* in phenylalanine-, arginine-, or tyrosine- limiting conditions. (A, B, C)** Confluent HFF monolayers in 96-well plates were prestarved for 24 hours in D1 complete media serially diluted in D1 missing phenylalanine (A), arginine (B), or tyrosine (C) to create D1 with concertation gradients for the experimental amino acid before infecting with 2,000 WTnLuc parasites per well with 3 technical replicates per dilution. Four hours post invasion, the media was changed keeping the dilution series, and the first reading was taken. The rest of the samples grew for 4 days at 37 °C in 5% CO_2_. Readings were normalized with the 4 hours post-infection infection readings and plotted as a percentage of growth as the complete media. A non-linear fit was used to determine the EC_50_. The dots are the mean growth with SD represented by the error bars. *n*=3 biological replicates.
